# *Lactiplantibacillus plantarum* LM1001 Supplementation Attenuates Muscle Atrophy and Function Decline in Aged Mice

**DOI:** 10.3390/nu17193156

**Published:** 2025-10-04

**Authors:** Jacques Karekezi, Hwajin Kim, Theodomir Dusabimana, Tatang Aldi Nugroho, Edvard Ntambara Ndahigwa, Yoon Ju So, Juil Kim, Tae-Rahk Kim, Minn Sohn, Ji Miao, Yuseok Moon, Sang Won Park

**Affiliations:** 1 Department of Pharmacology, Institute of Medical Sciences, College of Medicine, Gyeongsang National University, Jinju 52727, Republic of Korea; jacqueskarekezi@gmail.com (J.K.); hwajin1@gmail.com (H.K.); odomy2020@gmail.com (T.D.); aldinugroho178@gmail.com (T.A.N.); ndahigwaedvard@gmail.com (E.N.N.); 2 Department of Convergence Medical Sciences, Graduate School, Gyeongsang National University, Jinju 52727, Republic of Korea; 3 Microbiome R&D Center, Lactomason Co., Ltd., Jinju 52840, Republic of Korea; yjso@lactomason.com (Y.J.S.); trkim@lactomason.com (T.-R.K.); ms@lactomason.com (M.S.); 4 Department of Data Science and Convergence Medical Sciences, Pusan National University, Busan 46241, Republic of Korea; 1022myths@hanmail.net; 5 Division of Endocrinology, Boston Children’s Hospital, Boston, MA 02115, USA; ji.miao@childrens.harvard.edu

**Keywords:** aging, gut microbiota, muscle atrophy, myogenesis, probiotics

## Abstract

**Background/Objectives**: Aging and metabolic disorders are associated with a decline in muscle function, referred to as age-related sarcopenia. The underlying mechanisms of sarcopenia include cellular senescence, imbalanced protein homeostasis, accumulation of oxidative and inflammatory stressors, and mitochondrial dysfunction. Probiotic supplementation improves the gut microbiome and enhances muscle function via the gut–muscle axis. However, details of molecular mechanisms and the development of an appropriate treatment are under active investigation. **Methods**: We have examined the effects of *Lactiplantibacillus plantarum* LM1001, a probiotic that reportedly improves the digestibility of branched-chain amino acids in myocyte cultures, but exactly how it contributes to muscle structure and function remains unclear. **Results**: We show that aged mice (male C57BL6/J) fed a high-fat diet (HFD) exhibit weak muscle strength, as reflected by a reduction in grip strength. LM1001 supplementation increases muscle strength and restores myofibril size, which has been altered by HFD in aged mice. Expression of myogenic proteins is increased, while protein markers for muscle atrophy are downregulated by LM1001 treatment via the IGF-1/Akt/FoxO3a pathway. LM1001 improves gut microbiota that are altered in aged HFD-fed mice, by increasing their abundance in beneficial bacteria, and efficiently maintains the epithelial lining integrity of the large intestine. **Conclusions**: We conclude that LM1001 supplementation serves a beneficial role in patients suffering from sarcopenia and metabolic disorders, improving their muscle function, gut microbiota, and intestinal integrity.

## 1. Introduction

Sarcopenia is an age-related progressive loss of skeletal muscle mass, strength, and function, and is accompanied by physical disability and poor quality of life [1]. Muscle mass decreases with age, typically at a rate of 3–8% every 10 years after the age of 30; the changes are more pronounced and accelerate around age 60 [2]. Sarcopenia was diagnosed as a disease by the World Health Organization in 2016 [3], and classified in South Korea with a diagnosis code in 2021. Sarcopenia was estimated to affect 10–16% of the elderly worldwide, and the prevalence determined by the Asian Working Group for Sarcopenia (AWGS, 2014) criteria is 5.5–25.7% [4]. According to the European Working Group on Sarcopenia in Older People (EWGSOP2), the prevalence is 3.2–26.3% [5,6]. Sarcopenic obesity is the loss of muscle mass and strength, which often occurs in 60–65-year-old patients with increased body fat suffering from metabolic syndrome; the prevalence of sarcopenia is even higher, ranging from 18% in diabetic patients [7]. Risk factors for sarcopenia include cardiovascular and metabolic diseases associated with diabetes, hypertension, and hyperlipidemia. In elderly patients, metabolic imbalances and digestive dysfunction may be additional risk factors. Sarcopenic obesity is a new class of obesity in older adults in which low skeletal muscle mass is coupled with high levels of adiposity [8,9].

Current treatment of sarcopenia typically involves resistance exercise and protein intake containing leucine-rich essential amino acids to improve muscle mass, strength, and functional performance in elderly patients [10]. Regulators of muscle protein synthesis or breakdown have become direct targets of drug development for sarcopenia [11]. Treatment with growth hormone, such as insulin-like growth factor (IGF-1) [12], or androgen hormone, increases muscle mass and strength by regulating proliferation, myogenic differentiation, and anabolic protein metabolism [12]. Currently, use of selective androgen receptor modulators is in clinical trials, and reportedly shows improvement in total lean body mass, physical function, and insulin resistance [13]. However, in the case of senile sarcopenia, muscle pain or muscle decomposition are serious side effects. Therefore, there is a need to better understand the molecular mechanisms underlying muscle atrophy and regeneration to maintain muscle mass and function.

The composition of gut microbiota may determine skeletal muscle function by modulating systemic inflammation and insulin sensitivity, and may link the gut metabolic dysregulation to muscle atrophy or dysfunction, involving microbial metabolites such as oxidants, cytokines, or short-chain fatty acids [14]. Probiotic supplementation has been shown to attenuate age-related sarcopenia via the gut–muscle axis in senescence-accelerated mice [15]. This may be achieved by producing microbial metabolites with beneficial antioxidative and anti-inflammatory properties. However, the exact molecular mechanisms have not been elucidated, and further studies are needed on molecular factors that regulate intestinal, systemic inflammation, and/or metabolism, and their correlation to muscle atrophy and regeneration.

In this study, we used *Lactiplantibacillus plantarum* LM1001, which has been shown to improve protein digestibility and promote myogenesis in cultured myotubes [16], and explored further its effect on a mouse model of sarcopenia. This study demonstrates that LM1001 supplementation restores hand grip strength and myofibrillar size in aging and/or high-fat diet (HFD)-induced sarcopenia, by regulating the expression of muscle atrophy and myogenic proteins through the IGF-1/Akt/FoxO3a pathway. LM1001 also improves gut microbial dysbiosis and restores the epithelial cell lining of the large intestine that is altered by HFD. LM1001 may play a beneficial role in patients with sarcopenia and/or metabolic syndrome, improving their muscle function, the gut microbiota, and intestinal integrity.

## 2. Materials and Methods

### 2.1. Animals

Male C57BL/6J mice (40-week-old) were purchased from HLB Biostep (Incheon, Republic of Korea) and maintained in the animal facility at Gyeongsang National University. Mice were kept in a controlled environment with a temperature of 22 ± 2 °C, humidity of 50 ± 10%, and a 12 h light/dark cycle and provided with water and standard chow ad libitum. Protocols for animal experiments were approved by the Institutional Board of Animal Research at Gyeongsang National University (GNU-230612-M0122), and experiments were performed according to the NIH Guidelines for Care and Use of Laboratory Animals.

### 2.2. Probiotic Supplementation and Experimental Groups

LM1001 was obtained from the Department of Production, Lactomason (Jinju, Republic of Korea). Fifty-five-week-old mice were randomly divided into 4 groups: ND, ND+LM1001, HFD, and HFD+LM1001 (*n* = 9–12). Mice were fed with either a normal diet (ND) or HFD (60 Kcal% fat; Research Diets, Inc., New Brunswick, NJ, USA) with or without supplementation of LM1001 for 20 weeks. Food pellets contained a standardized probiotic dose of 5 × 10^9^ CFU per day. This dose was incorporated into 3 g of feed for the ND and 2 g for the HFD, resulting in microbial concentrations of 9.2 and 9.4 Log CFU/g, respectively. The average intake was reported as 9.3 ± 0.1 Log CFU/g of food. Food intake and body weight were recorded weekly. After 20 weeks of ND or HFD feeding with or without LM1001, all mice were sacrificed. Twelve-week-old mice fed with ND were also sacrificed as a young control group (*n* = 6). The gastrocnemius and quadriceps muscles, as well as the intestines, were collected and snap-frozen immediately in liquid nitrogen for storage at −80 °C, or fixed in 10% buffered formalin for histological analysis.

### 2.3. Grip Strength Test

Grip strength was measured at 12 weeks of age in young mice and 73 weeks of age in old mice. Mice were placed on a grid connected to a grip strength meter (Bioseb, Vitrolles, France) and allowed to hold the grid using only their forelimbs. The tail of each mouse was pulled parallel to the grid with constant force, toward the tester. A minimum of 5 measurements were made for each mouse at one-minute intervals, and the average value was calculated for each trial.

### 2.4. Hematoxylin & Eosin Analysis and Injury Scoring

Fixed tissues were embedded in paraffin, sectioned at 5 µm, and stained with hematoxylin and eosin (H&E, Sigma-Aldrich, St. Louis, MO, USA). Muscle atrophy: muscle fiber cross-sectional area (CSA) was analyzed by imaging on a CKX41 light microscope (Olympus, Tokyo, Japan); 5 stained images of 200× microscopic fields of each section. The area was quantified using the ImageJ software v.1.52a (NIH, Bethesda, MD, USA). Intestinal injury: scoring of disease severity was based on the Crypt damage and Ulceration. Crypt damage was scored as 0 = intact crypts; 1 = loss of the basal half; 2 = entire crypt loss; and 3 = confluent erosion. Ulceration was scored as 0 = absence of ulcer; 1 = one or two foci of ulcerations; 2 = three or four foci of ulcerations; and 3 = confluent or extensive ulceration.

### 2.5. Immunofluorescence (IF) Staining

Fixed tissues were embedded in paraffin and sectioned at 5 μm. Sections were deparaffinized, rehydrated, and immersed for antigen-retrieval in sodium citrate buffer (10 mM, pH 6.0) for 20 min. They were incubated overnight in a primary antibody against Pax7 (Proteintech Group, Rosemont, IL, USA, 20570-1-AP) at 4 °C, then in secondary antibody conjugated with Alexa Fluor^®^ 594 for 1 h at room temperature, and cover-slipped with ProLong Gold Anti-fade mounting solution containing DAPI (Thermo Fisher Scientific, Waltham, MA, USA). Images were captured on an Olympus Fluoview FV1000 confocal microscope (Olympus). The average fluorescence intensity was quantified using ImageJ software (NIH).

### 2.6. Western Blot Analysis of Muscle Tissues

Tissues were homogenized in ice-cold radio-immunoprecipitation assay (RIPA) buffer containing protease inhibitors (Thermo Fisher Scientific), sonicated, placed on ice for 30 min, and centrifuged. The supernatant was collected, and the protein concentration was measured using a Pierce™ BCA protein assay kit (Thermo Fisher Scientific). Protein lysates were separated by SDS-PAGE and transferred to PVDF membranes. Membranes were blocked with 5% skim milk or 3% BSA and incubated overnight with specific primary antibodies, Muscle ring-finger protein-1 (MuRF1), Myostatin, Myogenin (MyoG), and GAPDH (Invitrogen, Carlsbad, CA, USA), Muscle atrophy F-box gene (MAFbx, Atrogin-1), Myoblast determination protein 1 (MyoD1), Myogenic factor 5 (Myf5), and IGF-1 (Abcam, Cambridge, UK), Paired Box 7 (Pax7, Bioss, Woburn, MA, USA), Myosin heavy chain (MyHC, R&D Systems, Minneapolis, MN, USA), Desmin, *p*-FOXO3a, FOXO3a, *p*-AKT, and AKT (Cell Signaling Technology, Danvers, MA, USA) at 4 °C. The membranes were immersed in HRP-conjugated secondary antibodies (Bio-Rad, Hercules, CA, USA) at room temperature for 1 h and visualized using the ECL substrates system (Bio-Rad). The ChemiDoc XRS+ System (Bio-Rad) was used to evaluate the density of protein bands, and relative protein levels were quantified using ImageJ software (NIH).

### 2.7. Stool Sample Collection and DNA Extraction

Five to six stool pellets (100 mg) were collected for each mouse before sacrifice, stored at −80 °C prior to DNA extraction. Microbial DNA was extracted and purified from 100 mg of the fecal sample, using an Exgene Stool DNA mini kit (GeneALL, Seoul, Republic of Korea) according to the manufacturer’s instructions. The extracted DNA was quantified using Qubit fluorometer and a high-sensitivity dsDNA reagent kit (Invitrogen).

### 2.8. Amplification of the 16S rRNA Genes and Library Preparation

Total DNA samples from each group were pooled to prepare a 16S rRNA gene amplicon sequencing. The V4 region of 16S rRNA genes was amplified using a PCR primer set (533F, 5′-GTGCCAGCMGCCGCGGTAA-3′; 806R, 5′-GACTACHVGGGTWTCTAAT-3′) and Illumina sequencing adaptors (Illumina Inc., San Diego, CA, USA) using a KAPA HiFi HotStart Ready Mix (KAPA Biosystems, Wilmington, WA, USA) and standard cycling conditions. After purifying amplicons using AMPure^®^ XP beads (Agencourt Biosciences, Beverly, MA, USA), PCR products were validated for a library size using a BioAnalyzer 2100 (Agilent Technologies, Inc. Santa Clara, CA, USA) and subjected to indexing PCR using a Nextera XT Index Kit (Illumina, Inc.) and standard cycling conditions. The indexed PCR amplicons were purified using AMPure^®^ XP beads, verified for size using a BioAnalyzer, and quantified using the Qubit fluorometer. The quantified amplicons were diluted to 1 nM and pooled for sequencing on an Illumina iSeq platform (Illumina, Inc.), targeting 2 × 150 bp paired-end sequence reads.

### 2.9. Bioinformatic Data Processing

The procedure was based on the previously published method [17,18]. The resulting high-quality read pairs were analyzed through the Quantitative Insights into Microbial Ecology 2 (QIIME2, v2020.2) pipeline [19]. To enhance read quality, thirty-four bases of the reverse reads were truncated using the DADA2 algorithm [20]. The read pairs were then joined, denoised, dereplicated, and subjected to chimera removal. The processed data were then analyzed in terms of Amplicon Sequence Variants (ASVs). Taxonomic classification of representative 16S rRNA sequences was carried out using the QIIME2 naive Bayes classifier, which was trained on 99% operational taxonomic units (OTUs) and primer regions from the SILVA rRNA database (v138) [21]. A heatmap, representing the percentage abundance of the top 30 OTUs in any sample, was generated using the QIIME2R R package (v0.99.22).

### 2.10. In Vitro Cell Culture and Differentiation

Mouse myoblast C2C12 cells were cultured in DMEM (Welgene, Daegu, Republic of Korea) supplemented with 10% FBS (Gibco, Burlington, ON, Canada) and 1% penicillin-streptomycin (Gibco). At 80–90% confluency, cells were differentiated in the differentiation medium (DM), containing 2% horse serum and 1% of antibiotics, and treated with LM1001 (1 × 10^7^ CFU/mL) for 24 h. To induce atrophy, cells were stimulated with 100 μM of dexamethasone (Sigma-Aldrich) for 48 h after differentiation for 7 days. Cell morphology was visualized with a ZEISS microscope (ZEISS, Oberkochen, Germany). The average myotube diameter was measured from 40 myotubes per sample.

### 2.11. Western Blot Analysis of C2C12 Cells

C2C12 cells were lysed with RIPA buffer containing protease inhibitor, and the indicated protein expression was analyzed using Jess Simple Western capillary separation module (ProteinSimple, Bio-Techne, Minneapolis, MN, USA); relative expression levels were calculated using Compass Simple Western version 4.1.0 software (Bio-Techne).

### 2.12. Creatine Kinase Activity of C2C12 Cells

Creatinine kinase activity in cell lysates was analyzed using a Creatinine Kinase Activity assay kit (Sigma-Aldrich) according to the manufacturer’s instructions and was normalized against protein concentration.

### 2.13. Statistical Analysis

Statistical difference was determined using a two-tailed Student’s *t*-test to compare two groups or by one-way analysis of variance (ANOVA) followed by Bonferroni’s multiple comparison test for multiple groups. For the PCA group comparison, we used Hotelling’s T^2^ test, a multivariate statistical test that is the multivariate equivalent of a standard Student’s *t*-test. All statistical analyses were performed by GraphPad Prism 9 Software v.9.50 (GraphPad Software Inc., La Jolla, CA, USA). Data were expressed as means ± standard error of the mean (SEM). A *p* value < 0.05 was considered statistically significant.

## 3. Results

### 3.1. LM1001 Supplementation Improves the Muscle Strength in Aged Mice

**Figure 1:**
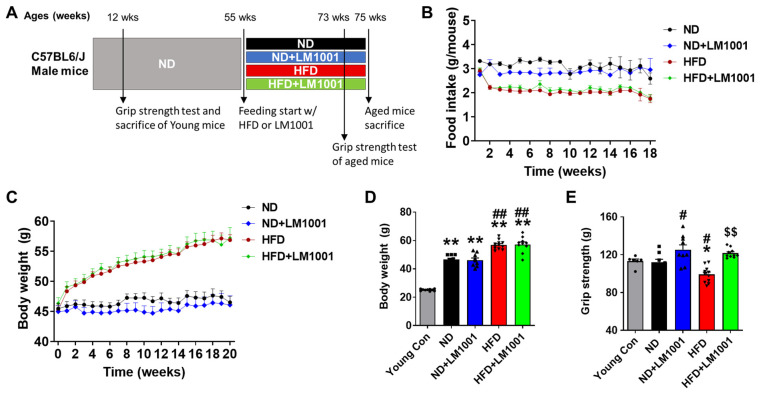
Effect of LM1001 on food intake, body weight, and muscle strength in aged mice. Aged mice (55 weeks old) were fed either ND or HFD for 20 weeks and then sacrificed. Healthy male 12-week-old mice fed ND were used as a young control group. (**A**) Experimental design of aged mice fed with ND or HFD for 20 weeks. (**B**) Food intake and (**C**) the body weight changes in aged mice for 20 weeks of ND or HFD feeding with LM1001 supplementation. (**D**) Body weight of mice at sacrifice. (**E**) Handgrip strength was measured at 18 weeks of HFD feeding (*n* = 6–12). Data are presented as the mean ± SEM. * *p* < 0.05, ** *p* < 0.01 versus young control group, ^#^ *p* < 0.05, ^##^ *p* < 0.01 versus ND group, and ^$$^ *p* < 0.01 versus HFD group.

We have established a mouse model of age-induced muscle atrophy. Aged mice (55-week-old) were either fed ND or HFD containing or lacking the probiotics LM1001 for 20 weeks to investigate its effect on muscle aging (Figure 1A). As expected, the HFD regimen increased body weight compared to the ND regimen, while LM1001 had no significant effect on ND or HFD food intake and body weight changes (Figure 1B–D). Grip strength is a simple and reliable measure of muscle function and physical ability. HFD feeding significantly reduced the hand grip strength of aged mice, but LM1001 supplementation significantly increased the grip strength of this aged group under both ND and HFD feeding conditions (Figure 1E). Collectively, our results indicate that probiotics LM1001 supplementation does not change body weight or food intake, but enhances muscle strength in aged mice.

### 3.2. LM1001 Supplementation Restores the Myofiber Size That Is Altered in Aged Mice

**Figure 2:**
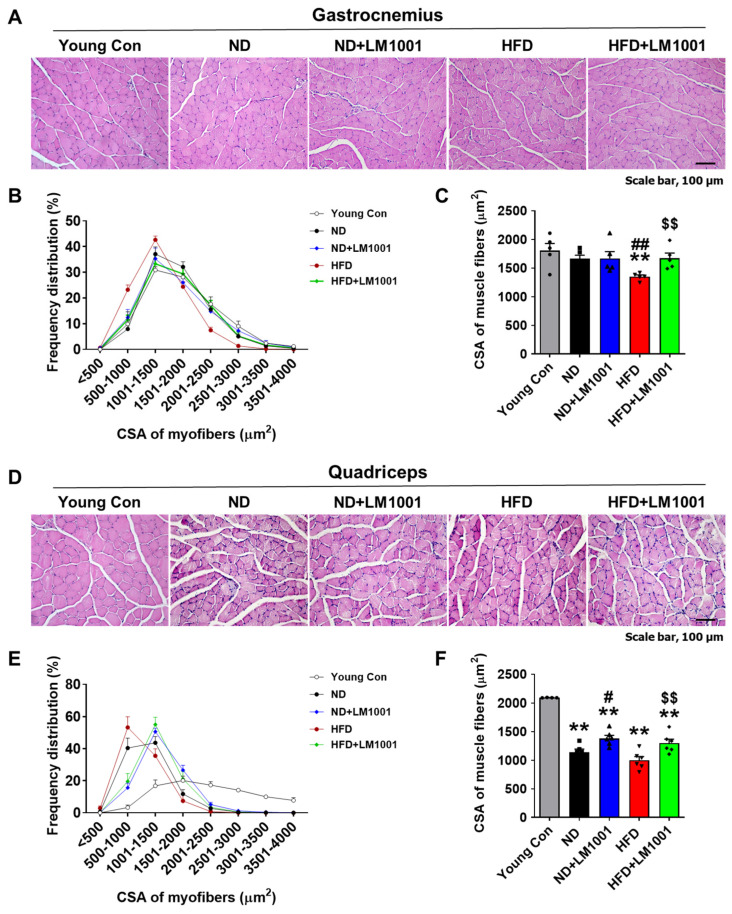
Effect of LM1001 on myofiber CSA in skeletal muscle of aged mice. Aged mice (55 weeks old) were fed either ND or HFD for 20 weeks and then sacrificed. Healthy male 12-week-old mice fed ND were used as a young control group. (**A**) Representative images of H&E staining, (**B**) Frequency distribution of CSA of myofibers, and (**C**) the mean CSA of myofibers from gastrocnemius sections. (*n* = 5). (**D**) Representative images of H&E staining. (**E**) Frequency distribution of CSA of myofibers, and (**F**) the mean CSA of myofibers from quadriceps sections of aged mice (*n* = 4–5). Data are presented as the mean ± SEM. ** *p* < 0.01 versus young control group, ^#^ *p* < 0.05, ^##^ *p* < 0.01 versus ND group, and ^$$^ *p* < 0.01 versus HFD group. Scale bar, 100 μm.

To investigate the effect of LM1001 on the morphology of muscle tissue, we measured the CSA of myofibers on sections through the gastrocnemius muscle (Figure 2A). In HFD-fed aged mice (Figure 2B; red dots), the relative frequency (%) histogram of CSA was shifted to the right by LM1001, towards larger myofibers (green dots). Mean CSA levels were significantly decreased in HFD-fed mice compared to ND-fed mice, but levels were restored by LM1001 supplementation (Figure 2C). Next, we measured the CSA of myofibers on sections from the quadriceps muscles (Figure 2D). In ND or HFD-fed aged mice (Figure 2E; black and red dots) the relative frequency (%) histogram of CSA was shifted to the right by LM1001, towards larger myofibers (blue and green dots). Mean CSA levels were significantly decreased in ND or HFD-fed aged mice compared to young control mice, but levels were restored by LM1001 supplementation (Figure 2F). The data suggest that LM1001 supplementation mitigates muscle atrophy observed in aged mice fed with HFD.

### 3.3. LM1001 Supplementation Reduces Muscle Atrophy Induced by HFD in Aged Mice

**Figure 3:**
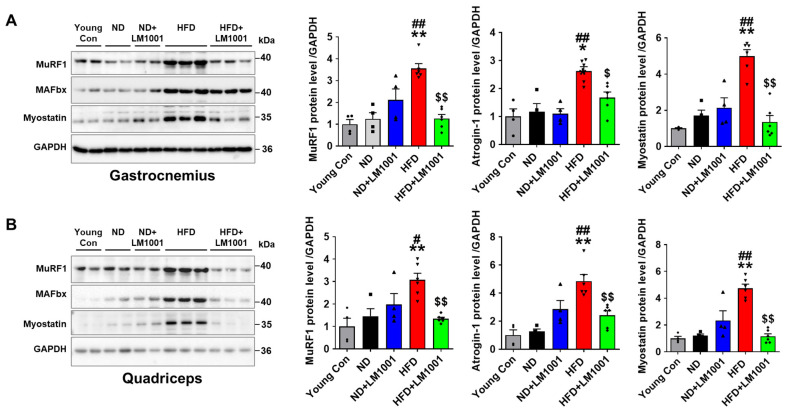
Effect of LM1001 on muscle atrophic proteins in skeletal muscle of aged mice. Aged mice (55 weeks old) were fed either ND or HFD for 20 weeks and then sacrificed. Healthy male 12-week-old mice fed ND were used as a young control group. (**A**) Gastrocnemius and (**B**) quadriceps tissue lysates were immunoblotted to determine muscle atrophic protein levels (MuRF1, Atrogin-1, and Myostatin), and the band intensities were quantified (*n* = 4–6). Data are presented as the mean ± SEM. * *p* < 0.05, ** *p* < 0.01 versus young control group, ^#^ *p* < 0.05, ^##^ *p* < 0.01 versus ND group, and ^$^ *p* < 0.05, ^$$^ *p* < 0.01 versus HFD group.

The effect of LM1001 on the expression of muscle atrophy proteins was investigated with the use of Western blot analysis in the skeletal muscle of mice. The levels of muscle atrophy proteins, MuRF1, Atrogin-1, and Myostatin, were significantly increased in gastrocnemius and quadriceps tissues of HFD-fed aged mice, compared to young and ND-fed aged mice; however, the levels were decreased by LM1001 (Figure 3A,B).

### 3.4. LM1001 Supplementation Promotes Myogenesis in the Skeletal Muscle of Aged Mice

**Figure 4:**
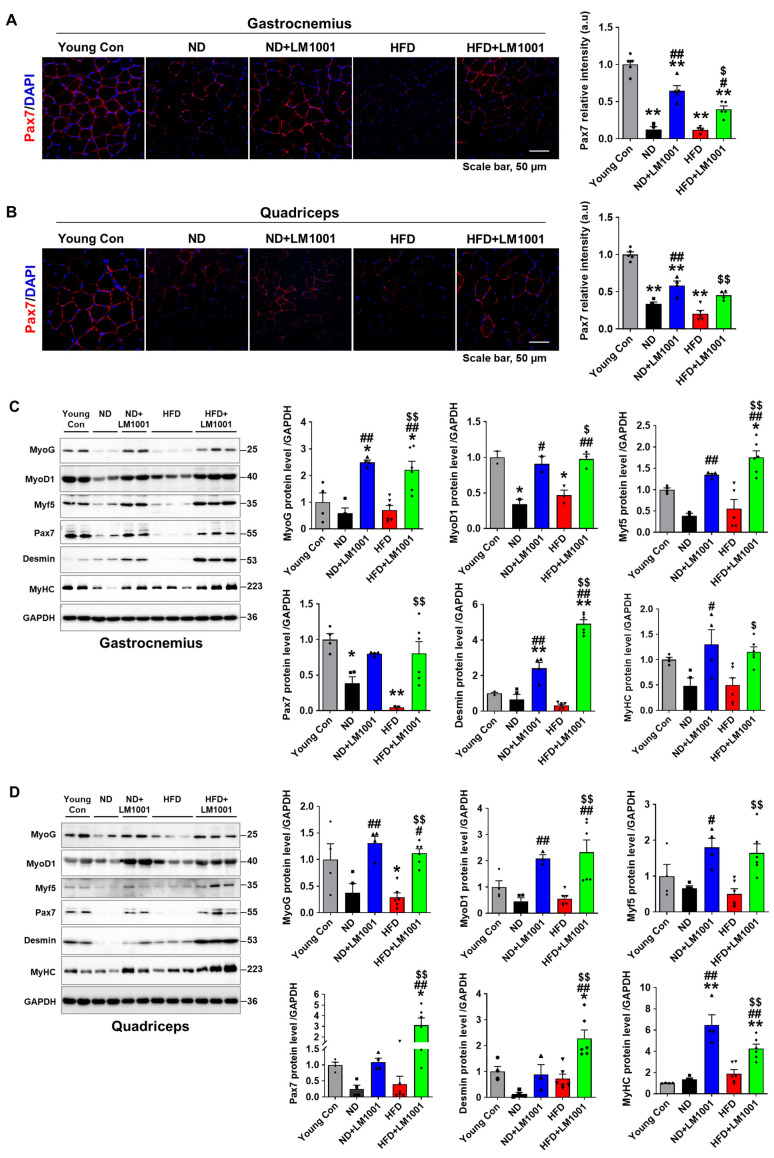
Effect of LM1001 on myogenic and muscle structural proteins in skeletal muscle of aged mice. Aged mice (55 weeks old) were fed either ND or HFD for 20 weeks and then sacrificed. Healthy male 12-week-old mice fed ND were used as a young control group. (**A**) Gastrocnemius and (**B**) quadriceps tissue sections were stained with Pax7 antibody. Representative immunofluorescence images of Pax7 and quantification of the relative intensity (a.u) were presented. (**C**) Gastrocnemius and (**D**) quadriceps tissue lysates were immunoblotted to determine myogenic protein (MyoG, MyoD1, Myf5, and Pax7) and muscle structural protein (Desmin and MyHC) levels, and the band intensities were quantified (*n* = 4–6). Data are presented as the mean ± SEM. * *p* < 0.05, ** *p* < 0.01 versus young control group, ^#^ *p* < 0.05, ^##^ *p* < 0.01 versus ND group, and ^$^ *p* < 0.05, ^$$^ *p* < 0.01 versus HFD group.

Immunofluorescence staining of Pax7 showed a reduction in mean signal intensity in aged mice fed with both ND and HFD compared to young mice, which was significantly increased by LM1001 supplementation (Figure 4A,B). We performed Western blot analysis on gastrocnemius and quadriceps muscle tissue lysates, and investigated the expression of myogenic transcription factors, MyoG, MyoD1, Myf5, and Pax7. In addition, we measured expression of Desmin, a muscle-specific intermediate filament protein [22], and MyHC, a molecular motor for muscle contraction [23]. Compared to young mice, expression of myogenic proteins was reduced in aged mice, but supplementation with LM1001 significantly restored these proteins in both ND and HFD-fed mice (Figure 4C,D). Together, these findings suggest that LM1001 protects against muscle atrophy and promotes muscle regeneration in normal-aged and HFD-fed mice.

**Figure 5:**
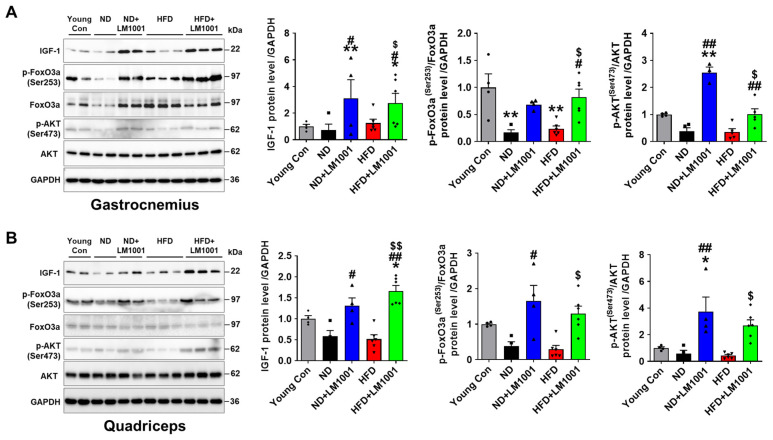
Effect of LM1001 on the IGF-1/Akt/FoxO3a pathway in skeletal muscle of aged mice. Aged mice (55 weeks old) were fed either ND or HFD for 20 weeks and then sacrificed. Healthy male 12-week-old mice fed ND were used as a young control group. (**A**) Gastrocnemius and (**B**) quadriceps tissue lysates were immunoblotted to determine the signal protein levels (IGF-1, p-Akt, Akt, p-FoxO3a, FoxO3a), and the band intensities were quantified (*n* = 4–6). Data are presented as the mean ± SEM. * *p* < 0.05, ** *p* < 0.01 versus young control group, ^#^ *p* < 0.05, ^##^ *p* < 0.01 versus ND group, and ^$^ *p* < 0.05, ^$$^ *p* < 0.01 versus HFD group.

The IGF-1 hormone plays a crucial role in growth and development, and is differentially regulated in aged versus young individuals [24]. In skeletal muscle, IGF-1 increases myogenesis through the PI3K/Akt/mTOR pathways and also inhibits muscle atrophy via Foxo3a-mediated ubiquitination. We used Western blot analysis to delineate the effect of LM1001 on the IGF-1/Akt/FoxO3a pathway. LM1001 significantly increased the levels of IGF-1 as well as of phosphorylated Akt (Ser473) and FoxO3a (Ser215) in both ND- and HFD-fed aged mice, leading to the downregulation of MuRF1 and Fbx32, which are responsible for muscle atrophy (Figure 5A,B). We thus conclude that LM1001 reduces muscle atrophy by activating the IGF-1/Akt/FoxO3a pathway in the skeletal muscles.

### 3.5. LM1001 Supplementation Improves the Gut Microbiota Altered in Aged HFD-Fed Mice

**Figure 6:**
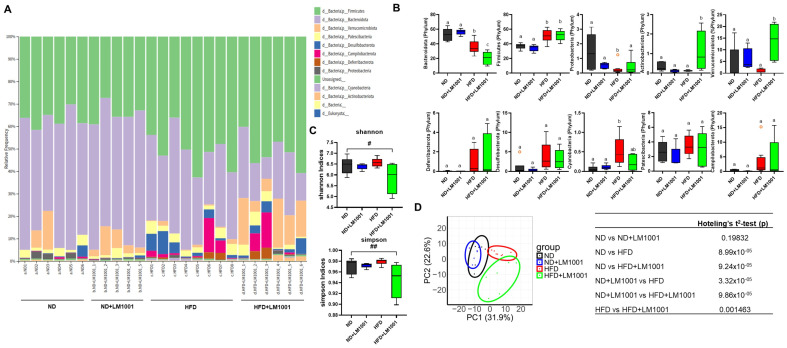
Effects of LM1001 on the abundance and diversity of gut bacteria in the feces of aged mice. Aged mice (55 weeks old) were fed either ND or HFD for 20 weeks, and then the feces were collected (*n* = 5–8). (**A**) A bar graph of bacterial composition at the phylum level from the feces of each mouse. (**B**) Specific phylum comparisons between groups. The results are shown as a plot with Tukey whiskers, and different letters above each bar represent significant differences between groups (*n* = 5–8, *p* < 0.05). (**C**) Gut bacterial alpha diversity was estimated by Shannon or Simpson indices (*n* = 4–6). Data are presented as the mean ± SEM. ^#^ *p* < 0.05, ^##^ *p* < 0.01 versus ND group. (**D**) Gut bacterial beta diversity was estimated by Principal coordinates analysis corresponding to the Bray–Curtis dissimilarity index with differential contribution factors. Different letters of the clusters represent significant differences between groups by Hotelling’s T^2^ test (lower table, *p* < 0.05).

To investigate how LM1001 influences the gut microbial community in aged mice, we performed 16S rRNA sequencing and compared the relative abundance of specific bacterial taxa across experimental groups (Figure 6). Administration of LM1001 to HFD–fed mice produced a pronounced shift in gut microbiota composition, characterized by a significant increase in the abundance of Verrucomicrobiota and Actinobacteriota, accompanied by a marked decrease in Bacteroidota under HFD conditions (Figure 6A,B). Verrucomicrobiota, which include beneficial species such as *Akkermansia muciniphila*, are widely recognized for their ability to strengthen the intestinal barrier, promote tight junction protein expression, and exert anti-inflammatory and metabolic benefits by modulating host immune responses and mucus layer thickness [25,26]. Similarly, members of the Actinobacteriota phylum, notably the genus *Bifidobacterium*, are known to produce short-chain fatty acids (SCFAs) such as acetate, enhance gut epithelial integrity, regulate immune balance, and inhibit the overgrowth of pathogenic bacteria [27]. In contrast, Bacteroidota, a phylum typically associated with fiber fermentation, SCFA production, and metabolic homeostasis, exhibited a significant reduction in abundance in HFD-fed mice, suggesting that HFD feeding disrupts beneficial microbial populations and compromises host metabolic health [28,29]. Alpha diversity analysis further revealed a remarkable reduction in microbial diversity in the HFD by LM1001 supplementation (Figure 6C). Beta diversity analysis supported this observation, showing that the microbial communities in the HFD group were distinctly clustered and also clearly separated by LM1001 (Figure 6D). Taken together, these findings indicate that LM1001 supplementation mitigates HFD-induced dysbiosis by selectively enriching microbial taxa with anti-inflammatory and barrier-protective properties, while an HFD alone promotes a decline in health-associated bacteria such as Bacteroidota.

### 3.6. LM1001 Supplementation Reduces the Large Intestinal Injury Induced by HFD in Aged Mice

**Figure 7:**
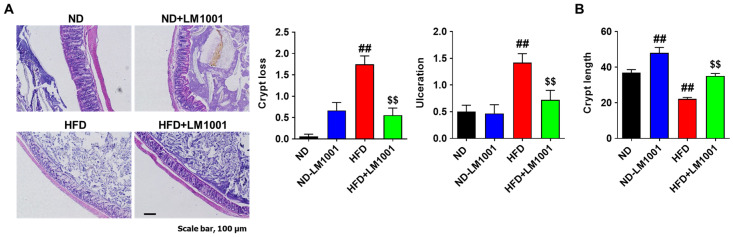
Effects of LM1001 on the large intestinal injuries in mice. Aged mice (55 weeks old) were fed either ND or HFD for 20 weeks and then sacrificed. Large intestine tissues were processed for H&E staining. The intestinal injury was scored as shown in crypt loss and ulceration (**A**), and the crypt lengths (**B**) of the large intestine were measured. Data are presented as the mean ± SEM. ^##^*p* < 0.01 versus ND group, and ^$$^ *p* < 0.01 versus HFD group.

To investigate the effect of LM1001 on the intestinal injury, we performed H&E staining of the large intestines and scored histologically based on two damage indices: crypt loss and ulceration. LM1001 supplementation reduced the levels of crypt loss and ulceration in HFD-fed mice (Figure 7A). LM1001 treatment showed a slight increase in the level of crypt loss, but no change in ulceration in ND-fed mice. To assess the structural changes in the large intestines, we measured the lengths of crypts and found that LM1001 significantly increased the lengths in both ND and HFD-fed mice (Figure 7B), suggesting LM1001 contributes to the restoration of the epithelial structure and function of the large intestine. Taken together, LM1001 improves the gut microbiota altered in aged HFD-fed mice and reduces the large intestinal injury in aged mice.

### 3.7. LM1001 Promotes Myogenic Differentiation and Reduces Dexamethasone-Induced Muscle Atrophy in C2C12 Cells

**Figure 8:**
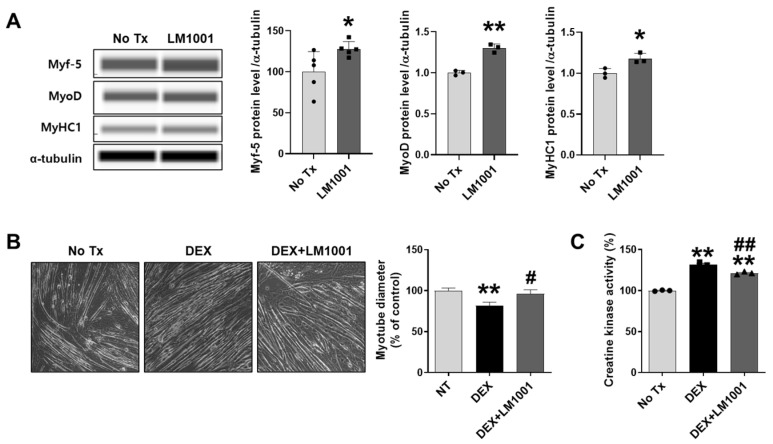
Effects of LM1001 on the myogenic mediators and muscle atrophy in C2C12 cells. (**A**) Cells (5 × 10^4^ cells/well) were seeded on a 24-well plate and cultured in growth medium for 3 days. At 90% confluency, the media was changed to differentiation medium with or without 1 × 10^7^ CFU/mL of LM1001. After 24 h, protein expression of myogenic markers was determined by immunoblotting. (**B**–**C**) Cells were differentiated in the differentiation medium with or without 1 × 10^7^ CFU/mL of LM1001 for 7 days, and the differentiated myotubes were stimulated with 100 μM of dexamethasone for 2 days to induce muscle atrophy. (**B**) Cell morphology was captured using an optical microscope (200× magnification), and the myotube diameters were measured from 40 individual myotubes. (**C**) The creatine kinase activity was measured in total proteins extracted from each group, and displayed as a percentage (%) of the No Tx control. All experiments were repeated at least three times. Data are presented as mean ± SEM. * *p* < 0.05, ** *p* < 0.01 versus no treatment (No Tx) control group. ^#^ *p* < 0.05, ^##^ *p* < 0.01 versus dexamethasone (DEX) group.

To directly investigate the effects of LM1001 on myoblasts, mouse myoblast C2C12 cells were treated with LM1001, and the myogenic and atrophic effects of LM1001 were examined. Cells were cultured for 24 h in DM containing LM1001 to determine how LM1001 influences myogenic differentiation. The protein levels for myogenic markers, Myf-5, MyoD, and MyHC1, were upregulated by LM1001 (Figure 8A). Next, to examine the effect of LM1001 on muscle atrophy, cells were differentiated for 7 days in DM containing LM1001, then treated for 2 days with dexamethasone (a synthetic glucocorticoid) to induce muscle atrophy. The diameter of myotubes was decreased by dexamethasone, but this reduction was significantly restored by LM1001 (Figure 8B). Consistent with this observation, the activity of creatine kinase, an indicator of muscle injury, was also reduced by LM1001 (Figure 8C). These results suggest that LM1001 prevents dexamethasone-induced muscle atrophy.

## 4. Discussion

We show that supplementation with probiotics LM1001 restores muscle grip strength and myofiber size in a mouse model of age-induced muscle atrophy, by regulating the expression of muscle atrophy and myogenic proteins through the IGF-1/Akt/FoxO3a pathway. LM1001 also improves gut microbial dysbiosis and restores the epithelial cell lining of the large intestine that is altered by HFD. LM1001 may play a beneficial role in patients with sarcopenia and/or metabolic syndrome, improving their muscle function, the gut microbiota, and intestinal integrity.

Fundamental aging mechanisms contributing to sarcopenia include cellular senescence, proteostasis imbalance, increased inflammation and oxidative stress, mitochondrial dysfunction, and fat accumulation, which collectively compromise the muscle’s ability to regenerate and maintain structural integrity [30]. Sarcopenia is strongly associated with muscle atrophy and a reduced capacity for muscle regeneration. Muscle regeneration encompasses muscle stem cell activation, proliferation, differentiation, fusion of myotubes, and formation of new myofibers; the processes are controlled by myogenic regulatory factors and specific growth hormones [31]. Probiotics supplementation has been implicated in improving muscle structure and function through their anti-inflammatory and antioxidant properties. In this study, we investigated the ability of LM1001 to attenuate muscle atrophy caused by aging and HFD. We have used myoblast cells in vitro and aged mice fed with ND or HFD, to show that LM1001 increases muscle strength and restores muscle myofibrillar structure, by directly regulating the expression of muscle atrophy and myogenic proteins.

Myogenesis plays a crucial role in muscle regeneration following injury and during muscle fiber formation. Paired box protein (Pax7), plus other transcription factors, is essential for activating muscle stem cells by regulating myogenic proliferation, differentiation, and fusion [32]. In this study, LM1001 supplementation increased the expression of myogenic transcription factors, MyoG, MyoD1, Myf5, Desmin, and MyHC in aged mice fed with ND or HFD, as well as Pax7. IGF-1 plays an important role in muscle development and growth. Binding of IGF-1 to the IGF-1 receptor activates PI3K/Akt, thereby mediating muscle proliferation via the mammalian target of rapamycin (mTOR) [33]. Activated Akt also inhibits muscle atrophy by inactivating FoxO3, a transcription factor that promotes muscle atrophy factors, MAFbx and MuRF1 [34,35,36]. In fact, IGF-1 levels and downstream signaling are suppressed in many chronic disease conditions, mediated by the combined effects of altered protein ubiquitination-protease system and autophagy [37]. The downstream kinases of PI3/Akt regulate cell proliferation and, at the same time, inhibit atrophy [38]. We show here that LM1001 supplementation also effectively regulates myogenesis and muscle atrophy through IGF-1/Akt/FoxO3a signaling pathways.

A recent study reports that suppression of sarcopenia by probiotics or muscle performance enhancement in rodents is not easily reproduced in humans [14]. This was possibly due to population diversity, limited availability of sample, and difficulties in accurately and reproducibly measuring muscle mass and function, which fail to attribute specific strains to sarcopenic phenotypes. Our findings reinforce the potential of probiotics but also emphasize the need to validate strain-specific effects in human populations. Recent advances in meta-omics approaches, meta-transcriptomics, meta-proteomics, metabolomics, and meta-genomics will enhance our functional understanding of the microbiome and lead to accurate prediction of host-microbiome interaction [39]. In humans, it is generally better to supplement probiotics in a specially colonized gut ecosystem, and balance this with prebiotics and/or protein intake, to enhance muscle performance and suppress sarcopenic phenotypes. Another approach is to create laboratory mice born to wild mice that have natural microbiota and pathogens, mimicking human immune responses; this could improve reproducibility, efficacy, and safety in biomedical applications [40].

Probiotics have a beneficial role in improving gut microbiota and intestinal integrity through the upregulation of paracrine hormone secretion and enhanced processing of metabolic derivatives such as branched amino acids [41]. Alpha diversity measures the diversity of microbial communities between individuals, while beta diversity measures the similarities or differences between two microbial communities [42,43]. Humans have co-evolved with microbial communities in the gut. Healthy children display functional and taxonomic differences from adults, where a sedentary lifestyle, westernized food intake, and medication strongly influence the composition of gut microbiota. In this study, HFD feeding markedly increased the abundance of bacteria that are pro-inflammatory and detrimental to intestinal integrity. LM1001 significantly influenced gut bacterial diversity in aged mice and improved their microbial dysbiosis.

Probiotics are beneficial for muscle homeostasis by producing SCFAs, especially acetate, propionate, and butyrate. In the senescent accelerated mice, supplementation of SCFAs enhances muscle mass and function [44,45]. Consistently, LM1001 supplementation provided a beneficial effect in sarcopenia, supported by previous reports of the impact of *Lactobacillus* on sarcopenia via the gut–muscle axis [46,47]. Study of how the gut–muscle axis is mediated by LM1001 and its SCFAs will extend our insight into their beneficial roles in relieving metabolic stress in muscles as well as other organs, and in enhancing overall body fitness. Our findings support the concept of a gut–muscle axis, whereby alterations in the gut microbiota increase SCFA production and promote muscle health. Consistent with previous work showing that SCFA supplementation enhances muscle mass and strength in aged mice [44], our results suggest that elevated SCFA levels can counteract sarcopenia. Importantly, human cohort studies have indicated that higher dietary fiber intake, which promotes the production of SCFAs, is positively associated with skeletal muscle mass and muscle strength in adults, as well as improved body composition in individuals with type 2 diabetes [48,49]. These findings suggest that the microbial and SCFA-mediated effects observed in our mouse model may also be relevant to humans. Moreover, although our intervention involved supplementation with LM1001, we recognize the potential for strain-specific effects within the *Lpb.* species. While LM1001 demonstrated beneficial effects on muscle outcomes in the present study, it is unlikely that all *Lpb.* strains will exert identical actions. Future studies employing strain-resolved approaches and functional validation will therefore be essential to identify the specific bacterial traits responsible for these muscle-protective effects.

Probiotics have beneficial roles in non-alcoholic fatty liver diseases (NAFLD). The severity of NAFLD correlates with components of the gut microbiome, such as Enterobacteriaceae, which affects de novo hepatic lipogenesis [50]. The compositions of gut microbiomes and metabolites in non-obese subjects are significantly different from those in obese NAFLD subjects and are associated with the pathogenesis of fibrosis. Administration of *Ruminococcus faecis* alleviates liver fibrosis in NAFLD mouse models, and restores gut homeostasis and gut barrier function [51]. Additionally, the interaction between gut microbiota (*Bacteroides vulgatus* and *Akkermansia muciniphila*) in obese subjects improves metabolic phenotypes and reduces hepatic steatosis by restoring the structure of the mucin layer and altered metabolites [52]. LM1001 also has a mild inflammatory effect on subjects on a normal diet, compared to those on HFD, wherein inflammation is reduced; this may be due to differences in microbiota or metabolite compositions, and requires further study. Overall, we argue that by switching beneficial bacteria over detrimental bacteria, intestinal structure and function can be improved. Delineating the beneficial metabolites or hormones secreted by bacteria could lead to potential therapeutic intervention to improve a healthy gut environment in the future.

Our study has some limitations. First, we did not measure circulating cytokines, myokines, or microbial metabolites (e.g., SCFAs) that could clarify the molecular mediators linking LM1001 to muscle and gut outcomes. Future studies are required to analyze plasma to determine the factors altered by aging. Second, the absence of a young control group limits our ability to determine whether LM1001 restores a youthful microbiota or primarily attenuates HFD-induced dysbiosis. Third, our analysis was limited to compositional measures of microbial diversity; functional assessments using metagenomics or metabolomics would strengthen causal inference. Finally, the study relied on a single probiotic strain and one animal model, restricting the generalizability of the findings. Future work should include multi-strain interventions, broader models of sarcopenia, and eventually well-controlled human trials.

## 5. Conclusions

In summary, supplementation with probiotics LM1001 restores muscle structure and function by regulating the expression of muscle atrophy and myogenic proteins through the IGF-1/Akt/FoxO3a pathway. LM1001 also improves gut microbial dysbiosis and restores the epithelial cell lining of the large intestine, which is altered by HFD. In addition, LM1001 may serve a beneficial role in patients with sarcopenia and/or metabolic syndrome by improving muscle function, gut microbiota, and intestinal integrity.

## Data Availability

The data presented in this study are available on request from the corresponding author, due to it is linked to ongoing additional research.
